# Menstrual and reproductive factors in relation to ovarian cancer risk

**DOI:** 10.1054/bjoc.2000.1596

**Published:** 2001-03

**Authors:** L Titus-Ernstoff, K Perez, D W Cramer, B L Harlow, J A Baron, E R Greenberg

**Affiliations:** Department of Community and Family Medicine, Dartmouth-Hitchcock Medical Center and the Norris Cotton Cancer Center, Lebanon, NH 03756; Department of Obstetrics and Gynecology, Obstetrics-Gynecology Epidemiology Center, Brigham and Women's Hospital, Boston, MA 02115; Department of Medicine, Dartmouth-Hitchcock Medical Center, Lebanon, NH 03756

**Keywords:** menstrual cycles, reproductive factors, ovarian cancer

## Abstract

We assessed menstrual and reproductive factors in relation to ovarian cancer risk in a large, population-based, case–control study. 563 cases in Massachusetts and New Hampshire were ascertained from hospitals and statewide tumour registries; control women (*n*= 523) were selected through random digit dialing and matched to case women by age and telephone sampling unit. We used multivariate logistic regression to evaluate factors in relation to risk of ovarian cancer and the major tumour histologic subtypes. Ovarian cancer risk was reduced among parous women, relative to nulliparous women (OR = 0.4; 95% CI = 0.3−0.6). Among parous women, higher parity (*P*= 0.0006), increased age at first (*P*= 0.03) or last (*P*= 0.05) birth, and time since last birth (*P*= 0.04) were associated with reduced risk. Early pregnancy losses, abortions, and stillbirths were unrelated to risk, but preterm, term, and twin births were protective. Risk was lower among women who had breast-fed, relative to those who had not (OR = 0.7; 95% CI = 0.5–1.0), but the average duration of breast-feeding per child was unrelated to risk (*P* for trend = 0.21). Age at menarche and age at menopause were unrelated to risk overall, although increasing menarcheal age was protective among premenopausal women (*P*= 0.02). Menstrual cycle characteristics and symptoms were generally unrelated to risk, although cycle-related insomnia was associated with decreased risk (OR = 0.5; 95% CI = 0.3–0.8). We found no association between the type of sanitary product used during menstruation and ovarian cancer risk. In analyses by histologic subtype, reproductive and menstrual factors had most effect on risk of endometrioid/clear cell tumours, and least influential with regard to risk of mucinous tumours. Overall, our findings offer some support to current hypotheses of ovarian pathogenesis, and show aetiologic differences among the tumour subtypes. © 2001 Cancer Research Campaign http://www.bjcancer.com


*British Journal of Cancer* (2001) **84**(5), 714–721
© 2001 Cancer Research Campaign
doi: 10.1054/ bjoc.2000.1596, available online at http://www.idealibrary.com on IDEAL

### Menstrual and reproductive factors in relation to ovarian cancer risk

**L Titus-Ernstoff[1], K Perez[1], DW Cramer[2], BL Harlow[2], JA Baron[1,3] and ER Greenberg[1,3]**

[1]Department of Community and Family Medicine, Dartmouth-Hitchcock Medical Center, and the Norris Cotton Cancer Center, Lebanon, NH 03756; [2]Obstetrics-Gynecology Epidemiology Center, Department of Obstetrics and Gynecology, Brigham and Women's Hospital, Boston, MA 02115; [3]Department of Medicine, Dartmouth-Hitchcock Medical Center, Lebanon, NH 03756

**Summary** We assessed menstrual and reproductive factors in relation to ovarian cancer risk in a large, population-based, case–control study. 563 cases in Massachusetts and New Hampshire were ascertained from hospitals and statewide tumour registries; control women ($n$ = 523) were selected through random digit dialing and matched to case women by age and telephone sampling unit. We used multivariate logistic regression to evaluate factors in relation to risk of ovarian cancer and the major tumour histologic subtypes. Ovarian cancer risk was reduced among parous women, relative to nulliparous women (OR = 0.4; 95% CI = 0.3−0.6). Among parous women, higher parity ($P$ = 0.0006), increased age at first ($P$ = 0.03) or last ($P$ = 0.05) birth, and time since last birth ($P$ = 0.04) were associated with reduced risk. Early pregnancy losses, abortions, and stillbirths were unrelated to risk, but preterm, term, and twin births were protective. Risk was lower among women who had breast-fed, relative to those who had not (OR = 0.7; 95% CI = 0.5–1.0), but the average duration of breast-feeding per child was unrelated to risk ($P$ for trend = 0.21). Age at menarche and age at menopause were unrelated to risk overall, although increasing menarcheal age was protective among premenopausal women ($P$ = 0.02). Menstrual cycle characteristics and symptoms were generally unrelated to risk, although cycle-related insomnia was associated with decreased risk (OR = 0.5; 95% CI = 0.3–0.8). We found no association between the type of sanitary product used during menstruation and ovarian cancer risk. In analyses by histologic subtype, reproductive and menstrual factors had most effect on risk of endometrioid/clear cell tumours, and least influential with regard to risk of mucinous tumours. Overall, our findings offer some support to current hypotheses of ovarian pathogenesis, and show aetiologic differences among the tumour subtypes. © 2001 Cancer Research Campaign http://www.bjcancer.com

**Keywords**: menstrual cycles; reproductive factors; ovarian cancer

The reduced risk of epithelial ovarian cancer associated with parity and oral contraceptive use suggests that pituitary and/or ovarian hormones or ovulatory events are important in the aetiology of these tumours (Cramer, 1986). Nevertheless, despite extensive study, the influence of menstrual and reproductive factors, with the exception of parity, remains uncertain. In this report, we evaluate menstrual and reproductive characteristics in relation to ovarian cancer risk overall, and in relation to the major tumour histologic subtypes. Our findings are considered in light of several current hypotheses regarding ovarian cancer pathogenesis. Briefly, the incessant ovulation hypothesis suggests that risk is increased by chronic post-ovulatory trauma to the epithelial surface of the ovary, and the tendency to form inclusion cysts (Fathalla, 1971; Casagrande et al, 1979). The gonadotrophin hypothesis proposes that excessive gonadotropin secretion and consequent increases in oestrogen stimulation lead to proliferation and malignant transformation of ovarian epithelium (Cramer, 1983). More recent hypotheses have suggested a role for chronic ovarian inflammation (Ness, 1999), androgens and progesterone (Risch, 1998), and the possibility that pregnancies reduce risk by clearing transforming cells from the ovaries (ovarian clearance) (Adami et al, 1994).

*Received 27 July 2000*
*Revised 3 November 2000*
*Accepted 3 November 2000*

*Correspondence to:* L Titus-Ernstoff

### METHODS

We conducted a population-based, case-control study of epithelial ovarian cancer in eastern Massachusetts (MA) and New Hampshire (NH). The methods used have been previously described (Cramer et al, 1998; Harlow et al, 1998). Briefly, 1033 potentially eligible case women (ages 20 to 74) were ascertained between May 1992 and March 1997 through state-wide cancer registries (NH, MA) and hospital tumour boards (MA). After exclusion of patients who had nonepithelial tumours ($n$ = 52), and those who had died ($n$ = 91), moved out of state ($n$ = 27), had no telephone ($n$ = 24), or did not speak English ($n$ = 14), 825 case women were eligible for the study. Physicians denied permission to contact 126 (14%) of these women, and 136 (16%) of women declined to participate. This analysis is based on 563 cases of epithelial ovarian cancer, including lesions of borderline malignancy.

Control women were identified primarily by random digit dialing (RDD) (Waksberg, 1978), and matched to case women by age (within 4 years) and telephone sampling unit. Of approximately 5400 residential households contacted, 10% declined to provide a household census, and 80% provided a census, but lacked a potentially eligible control subject. In the remaining 10% of households, a potentially eligible control was identified. 72% of the potential control women agreed to participate in the study. In MA, control women of age 60 or older were randomly chosen from Town Books, and matched to case women by age and precinct. Of 328 control women selected from Town Books, 21%

could not be reached, 18% were ineligible, and 30% declined to participate. Women who reported a bilateral oophorectomy were ineligible for participation. The present analyses are based on a total of 523 RDD and Town Book control women.

We conducted in-person interviews at the homes of case and control study participants. The questionnaire elicited demographic characteristics, lifestyle factors, dietary patterns, menstrual and reproductive history, and personal and family medical history. Reproductive variables of interest included parity, age at first birth, age at last birth, time elapsed since the last birth, total number of childbearing years (time elapsed between the first and last birth), and breast-feeding (ever/never, average duration in months per breast-fed child). We also evaluated specific birth outcomes, including abortion, miscarriage, stillbirth, preterm singleton live birth, full term singleton live birth, and twin birth in relation to ovarian cancer risk. Menstrual factors of interest were age at menarche, age at natural menopause, the number of months elapsed from menarche until menstrual cycle regularization (cycles predictable within 10 days), usual menstrual cycle length in days, usual number of days of menstrual bleeding, usual amount of menstrual bleeding, and sanitary products usually used between ages 20 and 40 (tampons, napkins). We evaluated menstrual symptoms, including usual level of menstrual pain/cramping, tension/mood swings, depression/inability to cope, sweats/hot flashes, headaches, insomnia, weight gain/bloating/swelling, back aches/muscle or joint pain, and breast pain, in relation to risk. Menstrual and reproductive factors were also assessed (using dichotomous cutpoints) in relation to the major tumour histologic subtypes (serous borderline, serous invasive, mucinous, and endometrioid/clear cell). In general, tumour type was determined by a review of the pathology report; for ambiguous diagnoses (5%), we obtained microscopy slides for review by our study pathologist.

We used unconditional logistic regression models to generate odds ratios and 95% confidence intervals for risk factors in relation to ovarian cancer occurrence, and in relation to the major tumour histologic subtypes (Breslow and Day, 1980). Variables were treated as continuous in likelihood ratio tests of linear trend when appropriate. The analyses considered only the exposures occurring prior to the date of diagnosis for case women and one year prior to interview for control women. We found no evidence of confounding by education, body mass index (kg/m$^2$), marital status, religion, family history of ovarian or breast cancer (in mother or sisters), history of infertility (unable to conceive for ≥2 years), history of tubal ligation, perineal talc use, or oral contraceptive use. We have therefore mainly presented results adjusted for age, state (MA, NH), and parity (0, 1, 2, 3, 4, ≥5). The analyses of twin births and adverse birth outcomes were adjusted for age, state, and the number of full term singleton live births (0, 1, 2, -≥3). Stratified analyses, generally adjusted for age, state and parity, were undertaken to evaluate potential effect modification by menopausal status (premenopausal, postmenopausal), and by oral contraceptive use (never, ever). Likelihood ratio tests were used to examine the statistical significance of interactions. Women with unknown menopausal status ($n$ = 171) were excluded from the analyses of age at menopause in relation to ovarian cancer risk, and from the analyses stratified by menopausal status.

### RESULTS

Case women were less likely than control women to have ever married, and more likely to report a family history of ovarian cancer (Table 1); they also reported lower levels of education, and less use of oral contraceptives.

Relative to nulliparous women, parous women had a statistically significant reduction of ovarian cancer risk (OR = 0.4; 95% CI = 0.3–0.6) (Table 2). Among parous women, increased parity was inversely associated with risk ($P$ for trend = 0.0006). Women with one birth had a 40% reduced risk; risk was reduced by 80% for those with 5 or more births.

In an analysis restricted to parous women, ovarian cancer risk decreased significantly with increasing age at first birth ($P$ for trend = 0.03) (Table 2). The finding pertained to uniparous women, and to women who had at least two births (data not shown). Stratified analyses indicated that the lower risk associated with later age at first birth (≥25 vs. <25 years) was observed only in women who had used oral contraceptives, and

**Table 1** Distribution of case and control women by select factors[a]

| Factor | Cases $n$ = 563 number (%) | Controls $n$ = 523 number (%) |
|---|---|---|
| Age | | |
| <30 | 29 (5.2) | 37 (7.1) |
| 30–39 | 78 (13.9) | 89 (17.0) |
| 40–49 | 159 (28.2) | 136 (26.0) |
| 50–59 | 126 (22.4) | 118 (22.6) |
| 60–69 | 118 (21.0) | 117 (22.4) |
| ≥70 | 53 (9.4) | 26 (5.0) |
| State | | |
| MA | 435 (77.3) | 411 (78.6) |
| NH | 128 (22.7) | 112 (21.4) |
| Marital status | | |
| Never married | 110 (19.5) | 61 (11.7) |
| Ever married | 453 (80.5) | 462 (88.3) |
| Education | | |
| Less than high school | 58 (10.3) | 30 (5.7) |
| High school graduate | 160 (28.4) | 141 (27.0) |
| Post-high school | 144 (25.6) | 152 (29.1) |
| College graduate | 201 (35.7) | 200 (38.2) |
| Family history of ovarian cancer[b] | | |
| No | 528 (94.3) | 505 (97.3) |
| Yes | 32 (5.7) | 14 (2.7) |
| Family history of breast cancer[b] | | |
| No | 500 (89.3) | 469 (90.2) |
| Yes | 60 (10.7) | 51 (9.8) |
| Religion | | |
| Non-Jewish | 509 (90.4) | 479 (91.6) |
| Jewish | 54 (9.6) | 44 (8.4) |
| Body mass index | | |
| <20 | 65 (11.5) | 55 (10.5) |
| 20.00–22.49 | 115 (20.4) | 134 (25.7) |
| 22.50–24.99 | 137 (24.3) | 124 (23.8) |
| 25.00–27.49 | 81 (14.4) | 78 (14.9) |
| 27.50–29.99 | 59 (10.5) | 50 (9.6) |
| ≥30 | 106 (18.8) | 81 (15.5) |
| Oral contraceptive use (months) | | |
| 0 | 312 (55.4) | 236 (45.1) |
| 1–12 | 98 (17.4) | 81 (15.5) |
| 13–60 | 80 (14.2) | 106 (20.3) |
| ≥61 | 73 (13.0) | 100 (19.1) |
| History of infertility[c] | | |
| No | 365 (80.6) | 376 (81.4) |
| Yes | 88 (19.4) | 86 (18.6) |
| History of tubal ligation | | |
| No | 488 (86.7) | 437 (83.6) |
| Yes | 75 (13.3) | 86 (16.4) |

[a] Variations in column totals are due to missing values. [b] Ovarian or breast cancer in mother or sister. [c] Among ever married women.

© 2001 Cancer Research Campaign

**Table 2** Distribution of case and control women by reproductive factors; odds ratios (OR) and 95% confidence intervals (CI) for the relationship with ovarian cancer risk.

| Reproductive factor | Cases number (%) | Controls number (%) | OR (95% CI)[a] |
|---|---|---|---|
| Parity | | | |
| Nulliparous | 185 (32.9) | 106 (20.3) | 1.0– |
| Parous | 378 (67.1) | 417 (79.7) | 0.4 (0.3–0.6) |
| 1 | 74 (13.1) | 63 (12.0) | 0.6 (0.4–0.9) |
| 2 | 132 (23.4) | 143 (27.3) | 0.4 (0.3–0.6) |
| 3 | 90 (16.0) | 107 (20.5) | 0.3 (0.2–0.5) |
| 4 | 50 (8.9) | 59 (11.3) | 0.3 (0.2–0.5) |
| ≥5 | 32 (5.7) | 45 (8.6) | 0.2 (0.1–0.4) |
| *P* for trend[b] | | | 0.0006 |
| Age at first birth[b] | | | |
| <20 | 46 (12.2) | 50 (12.0) | 1.2 (0.8–1.9) |
| 20–24 (referent) | 173 (45.8) | 180 (43.2) | 1.0– |
| 25–29 | 105 (27.8) | 130 (31.2) | 0.8 (0.6–1.1) |
| ≥30 | 54 (14.3) | 57 (13.7) | 0.9 (0.6–1.4) |
| *P* for trend | | | 0.03 |
| Age at last birth[b] | | | |
| <25 | 69 (18.3) | 53 (12.7) | 1.6 (1.0–2.5) |
| 25–29 (referent) | 119 (31.5) | 139 (33.3) | 1.0– |
| 30–34 | 110 (29.1) | 130 (31.2) | 1.0 (0.7–1.5) |
| ≥35 | 80 (21.2) | 95 (22.8) | 1.0 (0.6–1.5) |
| *P* for trend | | | 0.05 |
| Years since last birth[b] | | | |
| <10 | 42 (11.1) | 94 (22.5) | 0.6 (0.3–1.1) |
| 10–19 | 79 (20.9) | 74 (17.7) | 1.3 (0.8–2.0) |
| 20–29 (referent) | 140 (37.0) | 152 (36.5) | 1.0– |
| ≥30 | 117 (31.0) | 97 (23.3) | 1.0 (0.6–1.6) |
| *P* for trend | | | 0.04 |
| Breast-feeding[c] | | | |
| Never | 239 (63.2) | 223 (53.5) | 1.0– |
| Ever | 139 (36.8) | 194 (46.5) | 0.7 (0.5–1.0) |
| Average duration breast-feeding (months)[d] | | | |
| <3 | 43 (11.4) | 48 (11.5) | 0.9 (0.6–1.4) |
| 3–6 | 54 (14.3) | 76 (18.2) | 0.7 (0.5–1.1) |
| >6 | 42 (11.1) | 70 (16.8) | 0.7 (0.4–1.0) |
| *P* for trend[e] | | | 0.21 |

[a] OR for parity adjusted for age and state, all other ORs adjusted for age, state, and parity. [b] Among parous women. [c] Among women with at least one live birth. [d] Average duration (months) per breast-fed child, relative to not breast-feeding any child. [e] Among women who breast-fed.

in those whose ovarian cancer occurred before menopause; the statistical tests for interaction with oral contraceptive use ($P$ = 0.05) and menopausal status ($P$ = 0.002) were both significant. Further analyses indicated that the association between later age at first birth and reduced risk was confined to women who had used oral contraceptives and whose ovarian cancer was diagnosed before menopause (OR = 0.4; 95% CI = 0.2–0.7). Because of small numbers, we were unable to determine whether the reduced risk was more pronounced in women who had used oral contraceptives before their first birth, but the duration of oral contraceptive use was not correlated with age at first birth ($r = -0.04$; $P = 0.46$).

Risk decreased significantly with increasing age at last birth ($P$ for trend = 0.05) (Table 2). However, relative to a last birth between ages 25–29, there was no additional benefit associated with a last birth at age 30 or later. Among women with at least two births, age at first and last birth were moderately correlated ($r$ = 0.53, $P < 0.001$), and neither factor was significantly related to ovarian cancer risk when mutually adjusted. Risk increased with a related factor, the amount of time elapsed since the last birth ($P$ for trend = 0.04). There was no evidence that the total number of childbearing years was related to risk (for each year, OR = 1.00; 95% CI = 0.95–1.05).

Among women with at least one live birth, ever having breast-fed was associated with a statistically significant reduction in risk (OR = 0.7; 95% CI = 0.5–1.0) (Table 2). Breast-feeding for an average of 3–6 months, compared to never breast-feeding, was associated with a 30% decreased risk (OR = 0.7; 95% CI = 0.5–1.1). There was no additional benefit with a longer average duration of breast-feeding, and the test for trend was non-significant ($P$ = 0.21). The total duration of breast-feeding was also unrelated to risk ($P$ for trend = 0.34).

Most adverse pregnancy outcomes, including abortion, miscarriage, and stillbirth were unrelated to risk, although power was limited to evaluate the stillbirths (Table 3). A history of either a preterm (OR = 0.7; 95% CI = 0.4–1.0) or full-term singleton live birth (OR = 0.4; 95% CI = 0.3–0.6) was associated with a reduced risk. A history of twin birth appeared to be associated with

 *© 2001 Cancer Research Campaign*

**Table 3** Number and percent of case and control women reporting specific birth outcomes, odds ratios (OR), and 95% confidence intervals (CI) for the relationship with ovarian cancer risk

| Pregnancy outcome[b] | Cases number (%) | Controls number (%) | OR (95% CI)[a] |
|---|---|---|---|
| Abortion | 64 (11.4) | 56 (10.7) | 1.1 (0.7–1.6) |
| Miscarriage | 136 (24.2) | 139 (26.6) | 1.0 (0.7–1.3) |
| Stillbirth | 12 (2.1) | 10 (1.9) | 1.2 (0.5–2.9) |
| Preterm live birth | 41 (7.3) | 49 (9.4) | 0.7 (0.4–1.0) |
| Term live birth | 366 (65.1) | 403 (77.1) | 0.4 (0.3–0.6) |
| Twin birth | 11 (2.0) | 17 (3.3) | 0.6 (0.3–1.2) |

[a]Term live birth adjusted for age and state, all other pregnancy outcomes adjusted for age, state, and full term live singleton birth (0, 1, 2, ⩾ 3).
[b]Reference group is women who did not have this birth outcome.

decreased risk (OR = 0.6; 95% CI = 0.3–1.2), but the finding was not statistically significant. The findings in Table 3 were unchanged when further adjusted for all other birth outcomes, and when the analyses were restricted to gravid women (data not shown).

We also evaluated menstrual factors in relation to ovarian cancer risk. Menarcheal age was unrelated to risk overall ($P$ for trend = 0.38), although menarche at age 16 or more, relative to age 13, appeared to be associated with a decreased risk (Table 4). Age at menarche was inversely associated with risk among premenopausal women ($P$ for trend = 0.02), and the interaction between menopausal status and menarcheal age was statistically significant ($P$ = 0.002). Among premenopausal women, menarche at age 16 years or more, relative to before age 16, was associated with a marginally significant reduced risk (OR = 0.5; 95% CI = 0.2–1.0). Age at

**Table 4** Distribution of case and control women by menstrual factors[a]; odds ratios (OR) and 95% confidence intervals (Cl) for the relationship with ovarian cancer risk

| Menstrual factor | Cases number (%) | Controls number (%) | OR (95% CI)[b] |
|---|---|---|---|
| Age at menarche | | | |
| <11 | 36 (6.4) | 33 (6.3) | 1.1 (0.7–1.9) |
| 11–12 | 218 (38.8) | 198 (37.9) | 1.0 (0.7–1.3) |
| 13 (referent) | 175 (31.1) | 155 (29.7) | 1.0– |
| 14–15 | 108 (19.2) | 99 (19.0) | 1.0 (0.7–1.5) |
| ≥16 | 25 (4.4) | 37 (7.1) | 0.6 (0.4–1.1) |
| *P* for trend | | | 0.38 |
| Age at natural menopause | | | |
| <45 | 35 (15.5) | 28 (14.4) | 1.1 (0.6–2.0) |
| 45–49 | 69 (30.5) | 55 (28.4) | 1.2 (0.7–1.9) |
| 50–54 (referent) | 97 (42.9) | 92 (47.4) | 1.0– |
| ≥55 | 25 (11.1) | 19 (9.8) | 1.4 (0.7–2.7) |
| *P* for trend | | | 0.71 |
| Months until menstrual cycle regularization | | | |
| 0 (referent) | 461 (81.9) | 418 (79.9) | 1.0– |
| 1–12 | 18 (3.2) | 21 (4.0) | 0.8 (0.4–1.6) |
| 13–60 | 17 (3.0) | 15 (2.9) | 1.2 (0.6–2.4) |
| ≥61 | 18 (3.2) | 16 (3.1) | 1.1 (0.5–2.2) |
| never regular | 49 (8.7) | 53 (10.1) | 0.8 (0.6–1.3) |
| *P* for trend[c] | | | 0.65 |
| Menstrual cycle length (days) | | | |
| <27 | 74 (13.2) | 64 (12.2) | 1.0 (0.7–1.4) |
| 27–29 (referent) | 330 (58.8) | 289 (55.3) | 1.0– |
| ≥30 | 106 (18.9) | 118 (22.6) | 0.8 (0.6–1.1) |
| never regular | 51 (9.1) | 52 (9.9) | 0.9 (0.6–1.3) |
| *P* for trend[c] | | | 0.45 |
| Days of menstrual bleeding | | | |
| <4 | 52 (9.3) | 52 (9.9) | 0.9 (0.6–1.3) |
| 4 | 91 (16.2) | 100 (19.1) | 0.8 (0.5–1.1) |
| 5 (referent) | 243 (43.3) | 207 (39.6) | 1.0– |
| 6 | 71 (12.7) | 69 (13.2) | 0.9 (0.6–1.3) |
| ≥7 | 104 (18.5) | 95 (18.2) | 1.0 (0.7–1.4) |
| *P* for trend | | | 0.55 |
| Amount of menstrual bleeding | | | |
| light | 34 (6.1) | 34 (6.8) | 0.8 (0.5–1.3) |
| moderate (referent) | 294 (52.4) | 239 (47.7) | 1.0– |
| moderately heavy | 137 (24.4) | 143 (28.5) | 0.7 (0.6–1.0) |
| heavy | 96 (17.1) | 85 (17.0) | 0.9 (0.6–1.3) |
| *P* for trend | | | 0.50 |
| Sanitary product[d] | | | |
| pads (referent) | 282 (70.7) | 219 (67.8) | 1.0– |
| tampons | 117 (29.3) | 104 (32.2) | 0.9 (0.6–1.3) |

[a] Variations in column totals are due to missing values. [b] Adjusted for age, state, parity. [c] Among women whose menstrual periods became regular. [d] Women who reported using both types of products were excluded from this analysis.

**Table 5** Distribution of case and control women by menstrual symptoms[a], odds ratios (OR) and 95% confidence intervals (CI) for the relationship with ovarian cancer risk

| Menstrual symptom | Cases number (%) | Controls number (%) | OR (95% CI)[b] |
|---|---|---|---|
| Menstrual pain/cramping | | | |
| no pain (referent) | 148 (26.4) | 148 (28.3) | 1.0– |
| mild pain | 201 (35.8) | 209 (40.0) | 0.9 (0.7–1.3) |
| moderate/severe pain | 212 (37.8) | 166 (31.7) | 1.2 (0.8–1.6) |
| *P* for trend | | | 0.31 |
| Other menstrual symptoms[c] | | | |
| tension/mood swings | 303 (54.0) | 302 (60.3) | 0.8 (0.6–1.1) |
| depression/inability to cope | 115 (20.5) | 124 (24.8) | 0.8 (0.6–1.1) |
| sweats/hot flushes | 59 (10.5) | 45 (9.0) | 1.3 (0.8–1.9) |
| headaches | 175 (31.2) | 182 (36.3) | 0.8 (0.6–1.1) |
| insomnia | 46 (8.2) | 74 (14.8) | 0.5 (0.3–0.8) |
| weight gain/bloating/swelling | 385 (68.6) | 352 (70.3) | 1.0 (0.7–1.3) |
| backaches/muscle or joint pain | 261 (46.5) | 237 (47.3) | 1.0 (0.8–1.3) |
| breast pain | 286 (51.0) | 262 (52.3) | 1.0 (0.8–1.3) |

[a] Variations in column totals are due to missing values. [b] Adjusted for age state, and parity. [c] Number and percent of women reporting these symptoms, reference category is not reporting this symptom.

natural menopause was not related to ovarian cancer risk (*P* for trend = 0.71).

The amount of time elapsed between menarche and menstrual cycle regularization (*P* for trend = 0.65), menstrual cycle length (*P* for trend = 0.44), the number of days of menstrual bleeding (*P* for trend = 0.55), and the amount of menstrual bleeding (*P* for trend = 0.50) were unrelated to ovarian cancer risk (Table 4). We also found no indication that risk was influenced by the type of sanitary product used during menstruation.

Most menstrual symptoms were unrelated to risk (Table 5), but cycle-related insomnia was associated with a significantly decreased risk (OR = 0.5; 95% CI = 0.3–0.8).

We examined key reproductive and menstrual factors (using dichotomous cutpoints) in relation to the major histologic subtypes (Table 6). Parous women, compared to nulliparous women, had a strong (60%) and significantly reduced risk of all the tumour subtypes. Increasing parity was significantly associated with reduced risk of serous invasive (*P* for trend = 0.003) and endometrioid/clear cell tumours (*P* for trend = 0.002). An apparent inverse association between parity and serous borderline tumours (data not shown) was not statistically significant. There was no evidence, among parous women, that increasing parity was associated with risk of mucinous tumours.

Relative to a first birth before age 25, a first birth at a later age was associated with a significant (60%) reduced risk of serous borderline tumours, and a marginally significant (40%) decreased risk of endometrioid/clear cell tumours; increasing age at first birth was significantly associated with a decreased risk of these tumour types (Table 6). Increasing age at last birth was significantly associated with decreased risk of endometrioid/clear cell tumours (*P* for trend = 0.0009). Ever having breast-fed was associated with a significant (60%) reduction in risk of endometrioid/clear cell tumours; risk of this subtype also decreased with a longer average duration of breast-feeding (*P* for trend = 0.04). Breast-feeding was not related to risk of the other subtypes.

Menarche at age 16 or later, relative to before age 16, was associated with a significant (90%) decreased risk of endometrioid/clear cell tumours, but there was no evidence of linear trend, and menarcheal age was unrelated to other tumour subtypes. Age at menopause was not associated with risk of any tumour subtype.

**Table 6** Odds ratios[a] (OR) and 95% confidence intervals (CI) for the relation of select menstrual and reproductive factors to risk of the major tumor histologic subtypes

| Risk factor | Serous borderline (86 cases) OR (95% CI) | Serous Invasive (229 cases) OR (95% CI) | Mucinous (83 cases) OR (95% CI) | Endometrioid/clear cell (130 cases) OR (95% CI) |
|---|---|---|---|---|
| Parous[b] | 0.4 (0.3–0.7) | 0.4 (0.3–0.7) | 0.4 (0.2–0.7) | 0.4 (0.2–0.6) |
| *P* for trend[c] | 0.20 | 0.003 | 0.81 | 0.002 |
| Age ≥ 25 at first birth[c] | 0.4 (0.2–0.9) | 1.0 (0.6–1.4) | 0.9 (0.5–1.7) | 0.6 (0.4–1.0) |
| *P* for trend[c] | 0.02 | 0.52 | 0.65 | 0.006 |
| Age ≥ 30 at last birth[c] | 0.8 (0.4–1.6) | 1.0 (0.7–1.5) | 1.1 (0.6–2.0) | 0.7 (0.4–1.1) |
| *P* for trend[c] | 0.11 | 0.89 | 0.39 | 0.0009 |
| Ever breastfed[d] | 0.8 (0.4–1.6) | 1.0 (0.6–1.4) | 0.6 (0.3–1.2) | 0.4 (0.2–0.7) |
| *P* for trend[e] | 0.61 | 0.34 | 0.88 | 0.04 |
| Menarcheal age ≥ 16 | 1.0 (0.4–2.4) | 0.6 (0.3–1.3) | 1.3 (0.6–3.2) | 0.1 (0.0–0.8) |
| *P* for trend | 0.59 | 0.30 | 0.60 | 0.59 |
| Age at menopause <50 | 1.9 (0.5–7.6) | 0.9 (0.6–1.5) | 1.8 (0.7–4.6) | 0.9 (0.5–1.7) |
| *P* for trend | 0.76 | 0.41 | 0.32 | 0.48 |

[a] Variables (other than parous) adjusted for age, state, parity. [b] Adjusted for age, state; reference is nulliparous. [c] Among parous women. [d] Among women who had at least one live birth. [e] Average duration (months) per breast-fed child.

 *© 2001 Cancer Research Campaign*

### DISCUSSION

The strong and consistently observed risk reduction associated with parity provides, in part, the basis of most hypotheses regarding ovarian pathogenesis. It has been shown, however, that interruption of ovulation during pregnancy, lactation, and oral contraceptive use is inadequate to account for the magnitude of the observed decrease in ovarian cancer risk (Risch et al, 1983); similarly, it seems unlikely that the suspension of ovulation-induced inflammation or changes in hormone levels would fully account for the observed risk reduction. Moreover, none of the existing hypotheses adequately explains the substantial reduction of risk associated with one birth event. A full or near-term pregnancy may induce a permanent change in pituitary-mediated hormonal production, or cause a decidual reaction that decreases the susceptibility of the ovarian and pelvic surface epithelium to malignant transformation (Cramer, 1999).

In this study, late age at first birth was associated with reduced risk, although the effect was observed only in women who had used oral contraceptives and whose ovarian cancer was diagnosed before menopause. A few previous studies (Purdie et al, 1995), including the collaborative analysis of 12 population-based studies (Whittemore et al, 1992) also noted an inverse relationship between age at first birth and risk, but most found no association (for example, Risch et al, 1994), or an increased risk (for example, Negri et al, 1991). In contrast to previous studies (Risch et al, 1994; Salazar-Martinez et al, 1999), we also found a significant association between increasing age at last birth and reduced risk. Our evaluation of a related variable, the number of years since last birth, showed that risk increased with greater time elapsed since the woman's last birth. In contrast to a previous study (Godard et al, 1998), we found no evidence that the total number of childbearing years was associated with ovarian cancer risk.

Menstrual cycles occurring between ages 25 and 39 are most likely to be ovulatory (Harlow and Ephross, 1995), and pregnancies occurring between these ages have a greater potential to interrupt ovulatory cycles. Thus, our observation of reduced risk associated with later ages at first or last birth provides some support for hypotheses regarding incessant ovulation or ovarian inflammation. Pituitary secretion of gonadotropins generally increases during adulthood, but decreases during pregnancy; thus, the protective effects of later childbirth are also consistent with the gonadotropin hypothesis. The decreasing risk associated with later age at last birth is also consistent with the ovarian clearance hypothesis, and in particular with the notion that the protective 'clearance' effect of pregnancy is greater in older women, who are more likely to have premalignant ovarian epithelial cells (Adami et al, 1994).

Our finding that adverse birth outcomes, including pregnancy losses, were not significantly associated with ovarian cancer risk is consistent with most (for example, Hartge et al, 1989; Chen et al, 1992; Polychronopoulou et al, 1993), but not all previous studies (for example, Whittemore et al, 1988; Negri et al, 1991). The reduced risk observed in association with preterm, full term, or twin births is consistent with the protective effect of parity. Gonadotropins are generally higher among the mothers of dizygotic twins, relative to mothers of singletons (Thomas et al, 1998), but we were unable to distinguish dizygotic from monozygotic twin births.

In this study, and in most previous reports (Gwinn et al, 1990; Rosenblatt et al, 1993; Whittemore, 1993), breast-feeding was associated with reduced risk. Consistent with some previous studies (Rosenblatt et al, 1993; Whittemore, 1993; Salazar-Martinez et al, 1999), but not all (Gwinn et al, 1990), we found no relation between risk and duration of breast-feeding. Ovulatory cycles may resume in conjunction with supplemental feedings after the first few months of breast-feeding, perhaps accounting for the absence of a dose–response relationship. The reduction of risk associated with breast-feeding is consistent with hypotheses regarding incessant ovulation or ovarian inflammation, and with the hypothesis regarding gonadotropin secretion, which is slowed during breastfeeding (Harlow and Ephross, 1995).

We found little evidence that menstrual factors or symptoms, which may reflect ovulatory function and/or hormonal milieu, influence ovarian cancer risk. In this study, consistent with most previous studies (for example, Franceschi et al, 1991; Chen et al, 1992; Whittemore et al, 1992; Hankinson et al, 1995; Purdie et al, 1995; Salazar-Martinez, 1999), menarcheal age was unrelated to risk overall. However, our findings indicated that increasing age at menarche was associated with reduced risk among premenopausal women; similarly, at least two previous studies have found a stronger association between late menarcheal age and reduced risk among younger women (Parazzini et al, 1989; Whittemore, 1993). These findings may reflect the better recall of menarcheal age among younger/premenopausal women, although age at menarche is reported with reasonably good accuracy (Bean et al, 1979). Early menarche is associated with a more rapid onset of ovulatory cycles, and with the tendency to sustain higher levels of luteal phase estradiol and progesterone (Vihko and Apter, 1984). Thus, a protective effect of late menarche, observed here in premenopausal women, is consistent with hypotheses regarding incessant ovulation (Fathalla, 1971; Casagrande et al, 1979) and ovarian inflammation (Ness and Cottreau, 1999), but inconsistent with the hypothesis that higher levels of progesterone are associated with reduced risk (Risch, 1998). Similar to most previous reports (for example, Cramer et al, 1983; Hartge et al, 1988; Chen et al, 1992), we found no evidence of an association between age at natural menopause and ovarian cancer risk. The lack of an association between risk and age at natural menopause, which is recalled with reasonable accuracy (Bean et al, 1979), reduces the plausibility of hypotheses involving ovulatory events, or ovulation-associated ovarian inflammation.

In this study, as in most previous studies (Cramer et al, Chen 1983; et al, 1992; Purdie et al, 1995), ovarian cancer risk was not influenced by patterns or characteristics of the menstrual cycle. Although one previous study suggested an association between heavier menstrual bleeding and increased risk (Purdie et al, 1995), we found no effect for the duration or the heaviness of menstrual bleeding. While our results suggest that cycle characteristics are unrelated to risk, such factors are poorly recalled (Bean et al, 1979; Harlow and Ephross, 1995), and errors of self-report may have attenuated underlying effects. In our data, there was no association between the type of sanitary product used during menstruation and ovarian cancer risk.

In this study, consistent with previous reports (Cramer et al, 1983; McGowan et al, 1988; Chen et al, 1992; Purdie et al, 1995), menstrual symptoms were largely unrelated to ovarian cancer risk. However, we found preliminary evidence of reduced risk associated with insomnia occurring in conjunction with the menstrual cycle, which to our knowledge has not been previously reported. Oestrogen levels are likely to be lower, on average, in women who experience insomnia (Empson and Purdie, 1999). Thus, our

*© 2001 Cancer Research Campaign*

finding of decreased risk associated with insomnia, while preliminary, may reflect disparate hormonal milieus for case and control women.

Because of the large size of our study, we were able to evaluate menstrual and reproductive factors in relation to the major tumour histologic subtypes. Risk of all the tumour histologic subtypes was uniformly reduced for parous women, relative to nulliparas. However, we found no evidence that increasing parity was associated with decreased risk of mucinous tumours, whereas significant inverse trends were observed for serous invasive and endometrioid/clear cell tumours. Similar results were found in a case-control study conducted in Canada (Risch, 1996), and in a retrospective cohort study conducted in Norway (Kvale et al, 1988).

Our tumour subtype analyses showed that the influence of age at first birth was limited to serous borderline and endometrioid/clear cell tumours; age at last birth was associated only with endometrioid/clear cell tumours. No effects of age at first birth on risk of the tumour subtypes were observed in the Norwegian or the Canadian study. In our study, breast-feeding and average duration of breast-feeding were significantly associated only with endometrioid/clear cell tumours. In the Canadian study, the association between duration of breast-feeding and risk of endometrioid tumours was marginally significant, whereas a significant relationship was noted for serous invasive tumours (Risch, 1996). Breast-feeding was not evaluated in the Norwegian study.

In our data, increased age at menarche was not associated with decreased risk of any of the tumour subtypes. However, a menarcheal age of 16 years or more was associated with a dramatic reduction in risk of endometrioid/clear cell tumours. Menstrual factors were not assessed in the Canadian study, but the Norwegian study found significant effects for menarcheal age of 17 or more in relation to mucinous tumours. Consistent with the results of the Norwegian study (Kvale et al, 1988), we found no evidence that natural menopause was associated with any of the tumour subtypes.

### ACKNOWLEDGEMENTS

We thank Dr. Bernard Cole for statistical advice and personnel at both study centres for their commitment to this project. Most of all, we thank the study participants, whose generosity made this research possible. Supported by grant number R01 CA54419 from the National Cancer Institute.

### REFERENCES

Adami H, Hsieh C, Lambe M, Trichopoulos D, Leon D and Persson I (1994) Parity, age at first childbirth, and the risk of ovarian cancer. *Lancet* **344**: 1250–1254

Bean JA, Leeper JD, Wallace RB, Sherman BM and Jagger H (1979) Variations in the reporting of menstrual histories. *Am J Epidemiol* **109**: 181–185

Booth M, Beral V and Smith P (1989) Risk factors for ovarian cancer: a case-control study. *Brit J Cancer* **60**: 592–598

Breslow NE and Day NE (1980) *Statistical Methods in Cancer Research*. Volume 1 – The Analysis of Case-Control Studies. *IARC Sci Pub* **32**: 5–338

Casagrande JT, Louie EW, Pike MC, Roy S, Ross RK and Henderson BE (1979) "Incessant ovulation" and ovarian cancer, *Lancet* **2**: 170–173

Chen Y, Pao-Chen W, Long J-H, Ge W-J, Hartge P and Brinton LA (1992) Risk factors for epithelial ovarian cancer in Beijing, China. *Int. J Epidemiol* **21**: 23–29

Cramer DW (1986) Ovary. In: *Cancer Epidemiology and Prevention*, D Schottenfeld and JF Fraumeni, Jr (eds) pp. 871–880. Philadelphia, PA: W.B. Saunders Press

Cramer DW (1999) *Epidemiology of sporadic ovarian cancer*. Helene Harris Memorial Trust 7th International Forum on Ovarian Cancer. Karolinska Institute, Stockholm, Sweden

Cramer DW and Welch WR (1983) Determinants of ovarian cancer risk. II, Inferences regarding pathogenesis. *J Natl Cancer Inst* **71**: 717–721

Cramer DW, Hutchinson GB, Welch WR, Scully RE and Ryan KJ (1983) Determinants of ovarian cancer risk. I Reproductive experiences and family history. *J Natl Cancer Inst*, **71**: 711–716

Cramer DW, Harlow BL, Titus-Ernstoff L, Bohlke K, Welch WR and Greenberg ER (1998) Over-the-counter analgesics and risk of ovarian cancer, *Lancet* **351**: 104–107

Empson JA and Purdie DW (1999) Effects of sex steroids on sleep. *Ann Med* **31**: 141–145

Fathalla MF (1971) Incessant ovulation – a factor in ovarian neoplasia? *Lancet* **2**: 163

Franceschi S, La Vecchia C, Booth M, Tzonou A, Negri E, Parazzini F, Trichopoulos D and Beral V (1991) Pooled analysis of 3 European case-control studies of ovarian cancer. II. Age at menarche and at menopause. *Int J Cancer* **49**: 57–60

Godard B, Foulkes WD, Provencher D, Brunet J-S, Tonin PN, Mes-Masson A-M, Narod SA and Ghadirian P (1998) Risk factors for familial and sporadic ovarian cancer among French Canadians: A case-control study. *Am Jour Obstet Gynecol*, **179**: 403–410

Gwinn ML, Lee NC, Rhodes RH, Layde PM and Rubin GL (1990) Pregnancy, breast feeding, and oral contraceptives and the risk of epithelial ovarian cancer. *J Clin Epidemiol* **43**: 559–568

Hankinson SE, Colditz GA, Hunter DJ, Willett WC, Stampfer MJ, Rosner B, Hennekens C and Speizer FE (1995) A prospective study of reproductive factors and risk of epithelial ovarian cancer. *Cancer* **76**: 284–290

Harlow SD and Ephross SA (1995) Epidemiology of menstruation and its relevance to women's health. *Epidemiol Rev* **17**: 265–286

Harlow BL, Cramer DW, Baron JA, Titus-Ernstoff L and Greenberg ER (1998) Psychotropic medication use and risk of epithelial ovarian cancer. *Cancer Epidemiol Bio Prev* **7**: 687–702

Hartge P, Hoover R, McGowan L, Lesher L and Norris HJ (1988) Menopause and ovarian cancer. *Am J Epidemiol* **127**: 990–998

Hartge P, Schiffman MH, Hoover R, McGowan L, Lesher L and Norris HJ (1989) A case-control study of epithelial orarian cancer. *Am Jour Obstet Gynecol* **161**: 10–16

Kvale G, Heuch I, Nilssen S and Beral V (1988) Reproductive factors and risk of ovarian cancer: a prospective study. *Int J Cancer* **42**: 246–251

La Vecchia C, Decarli A, Franchesci S, Regallo M and Tognoni G (1984) Age at first birth and the risk of epithelial ovarian cancer. *J Natl Cancer Inst* **73**: 663–666

McGowan L, Norris HJ, Hartge P, Hoover R and Lesher L (1988) Risk factors in ovarian cancer. *Eur J Gyn Oncol*, **9**: 195–199

Negri E, Franchesci S, Tzonou A, Booth M, La Vecchia C, Parazzini F, Beral V, Boyle P and Trichopoulos D (1991) Pooled analysis of 3 European case-control studies: I. Reproductive factors and risk of epithelial ovarian cancer. *Int J Cancer* **49**: 50–56

Ness RB and Cottreau C (1999) Possible role of ovarian epithelial inflammation in ovarian cancer. *J Natl Cancer Inst* **91**: 1459–1467

Parazzini F, La Vecchia C, Negri C and Gentile A (1989) Menstrual factors and the risk of epithelial ovarian cancer. *J Clin Epidemiol* **42**: 443–448

Polychronopoulou A, Tzonou A, Hsieh C-c, Kaprinis G, Rebelakos A, Toupadaki N and Trichopoulos D (1993) Reproductive variables, tobacco, ethanol, coffee, and somatometry as risk factors for ovarian cancer. *Int J Cancer* **55**: 402–407

Purdie D, Green A, Bain C, Siskind V, Ward B, Hacker N, Quinn M, Wright G, Russell P and Susil B (1995) Reproductive and other factors and risk of epithelial ovarian cancer: An Australian case-control study. *Int J Cancer* **62**: 678–684

Risch HA (1996) Differences in risk factors for epithelial ovarian cancer by histologic type. Results of a case-control study. *Am J Epidemiol*, **144**: 363–371

Risch HA (1998) Hormonal etiology of epithelial ovarian cancer, with a hypothesis concerning the role of androgens and progesterone. *J Natl Cancer Inst*, **90**: 1774–1786

Risch JA, Weiss NS, Lyon JL, Daling JR and Liff JM (1983) Events of reproductive life and the incidence of epithelial ovarian cancer. *Am J Epidemiol*, **117**: 128–139

Risch HA, Marrett LD and Howe GR (1994) Parity, contraception, infertility, and the risk of epithelial ovarian cancer. *Am J Epidemiol*, **140**: 585–597

Rosenblatt KA (1993) Thomas DB, and the WHO Collaborative Study of Neoplasia and Steroid Contraceptives. Lactation and the risk of epithelial ovarian cancer. *Internatl J Epidemiol* **22**: 192–197

Salazar-Martinez E, Lazcano-Ponce EC, Lira-Lira GG, Escudero-De los Rios P, Salmeron-Castro J and Hernandez-Avila M (1999) Reproductive factors of ovarian and endometrial cancer risk in a high fertility population in Mexico. *Cancer Res*, **59**: 3658–3662

 *© 2001 Cancer Research Campaign*

Thomas HV, Murphy MF, Key TJ, Fentiman IS, Allen DS and Kinlen LJ (1998) Pregnancy and menstrual hormone levels in mothers of twins compared to mothers of singletons. *Ann Human Biol* **25**: 69–75

Vihko R and Apter D (1984) Endocrine characteristics of adolescent menstrual cycles: impact of early menarche. *J Ster Biochem* **20**: 231–236

Waksberg J (1978) Sampling methods for random digit dialing. *J Am Stat* **73**: 40–46

Whittemore AS (1993) Personal characteristics relating to risk of invasive epithelial ovarian cancer in older women in the United States. *Cancer* **71**: 558–565

Whittemore AS, Wu ML, Paffenbarger RS, Jr, Sarles DL, Kampert JB, Grosser S, Jung DL, Ballon S and Hendrickson M (1988) Personal and environmental characteristics related to epithelial ovarian cancer. II. Exposures to talcum powder, tobacco, alcohol, and coffee. *Am J Epidemiol* **128**: 1228–1240

Whittemore AS, Harris R and Itnyre J (1992), and the Collaborative Ovarian Cancer Group. Characteristics relating to ovarian cancer risk: collaborative analysis of 12 US case-control studies. *Am J Epidemiol* **136**: 1184–1203

Wu ML, Whittemore AS, Paffenbarger RS, Jr, Sarles DL, Kampert JB, Grosser S, Jung DL, Ballon S, Hendrickson M and Mohle-Boetano J (1988) Personal and environmental characteristics related to epithelial ovarian cancer. I. Reproductive and menstrual events and oral contraceptive use. *Am J Epidemiol* **128**: 1216–1227

*© 2001 Cancer Research Campaign*
